# Comparative Outcomes of Percutaneous Needle Tenotomy Performed by a Podiatrist Versus an Orthopaedic Surgeon in the Management of Toe Ulcers

**DOI:** 10.1002/jfa2.70123

**Published:** 2026-02-08

**Authors:** Adrian Heald, Sangeeth Priyadarshan Veluchamy Rathakrishnan, Jessica Blong, Andrew Sharpe, Andy Gorman, Edward Jude, Mike Stedman, Adam Robinson, Neil D. Reeves, Edward Gee, Matthew Allen

**Affiliations:** ^1^ Endocrinology & Diabetes Salford Royal NHS Foundation Trust Salford UK; ^2^ University of Manchester Manchester UK; ^3^ Trauma and Orthopaedic Surgery Salford Royal NHS Foundation Trust Salford UK; ^4^ Department of Podiatry Salford Royal NHS Foundation Trust Salford UK; ^5^ Tameside and Glossop Integrated Care NHS Foundation Trust Ashton‐under‐Lyne UK; ^6^ Res Consortium Andover UK; ^7^ Medical School Faculty of Health and Medicine Lancaster University Lancaster UK

**Keywords:** cost, foot ulcer, healing, intervention, podiatrist, tenotomy

## Abstract

**Background:**

Foot ulceration is a debilitating and often disabling complication of diabetes mellitus, with negative prognostic associations in terms of morbidity and mortality. Percutaneous needle tenotomy (PNT) is increasingly recognized as a safe, minimally invasive procedure for treating tendon‐related deformities, including mechanical forefoot ulceration. This study evaluated clinical outcomes of needle flexor tenotomies performed by a podiatrist versus an orthopaedic surgeon.

**Methods:**

This service evaluation reviewed consecutive adult patients with foot ulceration who received needle tenotomy by a senior podiatrist, excluding those who underwent alternative procedures/amputation. Orthopaedic surgeon conducted tenotomy was the comparison group.

**Results:**

Podiatrist: 30 patients underwent needle tenotomy (total—31 feet) (23 patients had diabetes). Orthopaedic surgeon: 10 patients underwent needle tenotomy (total—12 feet). Median age was 71.5 years (range: 39.0–92.0), with 8 males/2 females. (8 patients had diabetes). The podiatrist‐led group was older and had higher HbA1c, greater proportion of smokers, larger ulcer size and longer ulcer duration pre‐intervention (median 54 vs. 20 weeks and *p* = 0.002) than the orthopaedic surgeon–led group. Despite these differences in disease severity, time to ulcer resolution (median 4.7 vs. 2 weeks, *p* = 0.119) and the rate of complete healing (86.7% vs. 100% and *p* = 0.556) did not differ significantly between groups.

**Conclusion:**

The lowest cost of the minor surgical foot procedure as a day case = £554 (€662) with this cost at least halved by conducting the procedure in a podiatry clinic. In conclusion, podiatrist‐led percutaneous needle tenotomy is a safe and effective intervention for foot ulcers, achieving healing outcomes comparable to an orthopaedic surgeon. We hope that the procedure can be adopted more widely.

## 1 Introduction

Foot ulceration is a debilitating and often disabling complication of diabetes mellitus, with negative prognostic associations in terms of morbidity and mortality [1]. Foot deformity can result in abnormally high forces through altered soft tissues with consequent development and maintenance of foot ulceration. In cases of diabetes‐related neuropathic ulceration of the toe apices or under the metatarsal heads, this ulceration may be amenable to treatment using simple surgical mechanical alterations. Early simple orthopaedic surgical intervention has the potential to provide dramatic and long‐lasting improvements in outcomes. Specifically percutaneous needle tenotomy (PNT) is increasingly recognized as a safe, minimally invasive procedure for treating tendon‐related deformities, including mechanical forefoot ulceration [2]. PNT is also known as tendon fenestration. The tendon is identified, and fenestrated was done up to 20 times with an 18‐ to 22‐gauge needle repeatedly [3].

Previously, we described that PNT was both safe and effective in treating forefoot ulceration in people with diabetes. Patients with toe ulcers present for an average of 5 months prior to intervention and achieved complete ulcer resolution within an average of 3.3 weeks [4].

Traditionally performed by orthopaedic surgeons [5], the procedure has been increasingly adopted by podiatrists. At our institution, we previously reported significant patient benefit, including reduced resource use and cost savings, when this straightforward intervention was performed by an orthopaedic surgeon. However, a comparative analysis on the outcomes based on the specialist type has not been published.

This study evaluated the clinical outcomes of needle flexor tenotomies performed by a podiatrist versus comparative cohort needle flexor tenotomies performed by an orthopaedic surgeon.

## 2 Methods

A retrospective service evaluation was conducted, including consecutive adult patients who presented with foot ulceration to the podiatry department of a tertiary care centre. The tenotomy procedure is now a routine practice in the department. Consecutive patients who underwent tenotomy performed by a senior podiatrist were included and compared with those who underwent tenotomy performed by a consultant orthopaedic surgeon. Written consent was obtained for the procedure and for data obtained in relation to clinical outcomes to be analysed and used for the purpose of clinical audit and publication.

All patient level data were stored within the secure database environment hosted by the Northern Care Alliance (NCA).

Peripheral neuropathy was defined as a vibration perception threshold (VPT) above 15V and peripheral vascular disease by the absence of both dorsalis pedis and tibialis posterior pulses on clinical examination of each limb.

Data for the orthopaedic surgeon–led and podiatrist‐led tenotomy were collected between 2nd January 2023 and 30th September 2024.

Patients were excluded if they underwent alternative percutaneous procedures, required amputation prior to assessment or lacked adequate follow‐up data to determine ulcer healing outcomes. Data from a separate cohort managed with orthopaedic tenotomy at our centre were used for comparison.

Ulcer swabs for culture and sensitivity were taken at presentation and at any point subsequently when infection was suspected. The SINBAD classification was used to define ulcer severity [6].

Exclusion criteria were deep or spreading infection and acute Charcot neuroarthropathy; tendon procedures were performed as part of a more complex surgical procedure including patients requiring Achilles and toe tendon procedures simultaneously.

Demographic, clinical and procedural variables were collected from hospital electronic patient records and included age at presentation, sex, number of affected feet, diabetes status, HbA1c levels at presentation, smoking status, presence of peripheral vascular disease, presence of peripheral neuropathy, ulcer surface area at presentation and duration of ulceration prior to intervention. The primary outcomes were time to complete ulcer resolution and the proportion of patients achieving complete healing. Secondary outcomes included 12‐month recurrence rates and incidence of amputation.

### 2.1 Informed Consent and Ethical Approval

It is a routine practice for this surgical intervention to be offered to eligible patients. Informed written consent was obtained for the surgical procedure. This was a service evaluation. Patients gave additional consent for their results to be anonymously used for service evaluation.

### 2.2 Statistical Analysis

Descriptive statistics were used to summarize the demographic, clinical and procedural characteristics, with continuous variables presented as means, medians and ranges. Categorical variables were expressed as frequencies and percentages.

For inferential analysis, categorical variables were compared using chi‐squared tests or Fisher's exact tests when expected cell counts were below five. Continuous variables were compared using Mann–Whitney U tests due to nonnormal distributions.

Given the small sample sizes and presence of tied ranks, exact two‐tailed *p*‐values were reported for the Mann–Whitney *U* tests. A *p*‐value of < 0.05 was considered statistically significant. Statistical analyses were performed using IBM SPSS Statistics, version 21.

## 3 Results

The mean SINBAD score [6] for the podiatrist‐led tenotomy was 2.8 (range 1–5) and for orthopaedic surgeon–led tenotomy was 2.7 (range 1–4).

### 3.1 Podiatrist‐Led Tenotomy Outcomes

In the podiatrist‐led tenotomy cohort, 30 patients underwent needle tenotomy for a total of 31 feet. The median age at presentation was 68.3 years (range: 52.6–88.0), with a predominant male distribution (23 male, 77%). In all, 23 patients had diabetes (2 patients had type 1 diabetes, and 21 patients had type 2 diabetes). The median HbA1c at presentation was 66 mmol/mol (8.2%), ranging 42–136 mmol/mol (6.0%–14.6%). 24 patients had peripheral neuropathy, and 8 patients had peripheral vascular disease (PVD). 10 patients had an ulcer in the first toe, and 20 patients had an ulcer in other toes (2–5). Ulcer characteristics revealed a median size of 0.24 cm^2^ (range: 0.01–2.7 cm^2^) and a median duration prior to intervention of 54 weeks (range: 13 to > 52 weeks). After tenotomy, the median time to ulcer resolution was 4.7 weeks (range: 1–18 weeks) (Table 1). Primary and secondary outcomes are shown in Table 2. The median time to ulcer resolution was 4.7 weeks, with 86.7% (26 patients) achieving complete healing. There was no direct complication for any patient. At the 12‐month follow‐up, 3 patients had ulcer recurrence, and 1 patient underwent amputation.

**TABLE 1:** Patient characteristics undergoing flexor tenotomy by podiatrists and orthopaedic surgeons.

Patient characteristics	Tenotomy by podiatrist	Tenotomy by orthopaedic surgeon	*p*‐value
Age at presentation (years)	Range 52.6–88.0	Range 39.0–92.0	*p* = 0.827
	Median 68.3	Median 71.5	
	Mean 69.4	Mean 70.8	
Sex	23 male	8 male	*p* = 1.0
	7 female	2 female	
Feet affected	31 feet	12 feet	NA
Characteristics	23 diabetes — 2 TID — 21 T2D 7 non‐diabetes	8 diabetes — 2 TID — 5 T2D — 1 pancreatic secondary 2 nondiabetes	*p* = 1.0
HbA1c at presentation mmol/mol (%)	Range 42–136 (6.0–14.6)	Range 15–68 (3.5–8.4)	*p* = 0.044
	Median 66 (8.2)	Median 44.1 (6.2)	
	Mean 68.7 (8.4)	Mean 45.3 (6.3)	
Smoking status	15 smokers	1 smokers	*p* = 0.032
	15 non‐smokers	9 nonsmokers	
Peripheral vascular disease	8 yes	2 yes	*p* = 1.0
	22 no	8 no	
Neuropathy	24 yes	9 yes	*p* = 0.656
	6 no	1 no	
Ulcer location	10 first toe (80% plantar, 20% dorsal)	3 first toe (70% plantar and 30% dorsal)	*p* = 1.0
	20 other toes (2–5)	7 other toes (2–5)	
	(55% apical, 45% dorsal)	(50% apical and 50% dorsal)	
Ulcer size at presentation (cm^2^)	Range 0.01–2.7	Range 0.01–0.28	*p* = 0.028
	Median 0.24	Median 0.08	
	Mean 0.52	Mean 0.12	
Ulcer duration prior to intervention (weeks)	Range 13–beyond 52	Range 5–beyond 52	*p* = 0.002
	Median 54	Median 20	
	Mean 85.9	Mean 21	

*Note:* Statistically significant at *p* < 0.05.

Abbreviations: NA: not applicable, T1D: type 1 diabetes, T2D: type 2 diabetes.

**TABLE 2:** Primary and secondary clinical outcomes following flexor tenotomy.

	Tenotomy by podiatrist	Tenotomy by orthopaedic surgeon	*p*‐value
Time to ulcer resolution (weeks)	Range 1–18	Range 1–11	*p* = 0.119
	Median 4.7	Median 2	
	Mean: 5.7	Mean 3.3	
Patients achieving complete ulcer resolution	26 (86.7%)	10 (100%)	*p* = 0.556
12‐month follow‐up rates:			
Recurrence	3	0	NA
Amputation	1	0	

*Note:* Statistically significant at *p* < 0.05.

Abbreviation: NA: not applicable.

### 3.2 Orthopaedic Surgeon–Led Tenotomy Outcomes

Data from the orthopaedic surgeon–led tenotomy cohort included 10 patients (12 feet) (Table 1). The median age was 71.5 years (range: 39.0–92.0), with 8 males and 2 females. Diabetes was present in 8 patients with median HbA1c at presentation of 44.1 mmol/mol (6.2%). Peripheral neuropathy was present in 9 patients and PVD in 2 patients. Of the group, 3 had an ulcer in the first toe and 7 had an ulcer in the other toes (2–5). Ulcer characteristics showed a median size of 0.08 cm^2^ (range: 0.01–0.28 cm^2^) and a median duration prior to intervention of 20 weeks (range: 5 to > 52 weeks). The median time to ulcer resolution post‐procedure was 2 weeks (range: 1–11 weeks). There was no direct complication for any patient. All patients achieved complete ulcer resolution, with no cases of ulcer recurrence or amputation at the 12‐month follow‐up.

### 3.3 Comparison Between the Orthopaedic Surgeon and the Podiatrist

Statistical analysis showed that the podiatrist‐led group had significantly higher HbA1c levels (median 66 mmol/mol vs. 44.1 mmol/mol and *p* = 0.044) (median 8.2% vs. 6.2% and *p* = 0.044), greater proportion of smokers (50% vs. 10% and *p* = 0.032), larger ulcer sizes (median 0.24 vs. 0.08 cm^2^ and *p* = 0.028) and longer ulcer duration before intervention (median 54 vs. 20 weeks, *p* = 0.002) than the orthopaedic surgeon–led group. Despite these differences in disease severity, the time to ulcer resolution (median 4.7 vs. 2 weeks and *p* = 0.119) and the rate of complete healing (86.7% vs. 100% and *p* = 0.556) did not differ significantly between groups (Table 2).

## 4 Discussion

This study demonstrates that tenotomy, when performed by a podiatrist, is an effective intervention for the management of foot ulcers, achieving an 86.7% complete healing rate. Despite patients in the podiatrist‐led group presenting with larger ulcers, longer ulcer durations and higher HbA1c levels factors traditionally associated with poorer healing outcomes, the healing rates were comparable to those observed in the orthopaedic surgeon–led group.

Reduction of local mechanical forces by lengthening or releasing tendons of the foot has the potential to reduce damaging vector forces through soft tissues and to improve local blood supply by reducing capillary compression on weight bearing, allowing the soft tissues to heal [7]. Tenotomy corrects the clawing of the toe and eliminates the pressure on the tip and the dorsum of the toe [8].

The orthopaedic surgeon–led group exhibited a 100% healing rate with a numerically shorter mean time to ulcer resolution. However, this group had patients with smaller ulcers, shorter ulcer durations and better glycaemic control at the baseline, which may have contributed to the more favourable outcomes. The lowest cost of the minor surgical foot procedure as day case is £554 (€662) [9]. This cost at least halved in a podiatry clinic, with appointments freed up for other patients because of the shorter healing time.

The comparable healing outcomes between the two groups, despite differing baseline characteristics, suggest that podiatrists can effectively perform tenotomies with outcomes like those achieved by orthopaedic surgeons. This has significant implications for multidisciplinary diabetic foot care, potentially expanding the scope of practice for podiatrists and improving access to timely surgical interventions in accordance with the NICE recommended best practice [10].

Inclusion criteria were strictly adhered to focus on procedures performed exclusively with the aim of cure. Any ulcers with suspected active clinical infection were excluded.

Limitations of the study are its retrospective design and the small sample size; plus, this was a single centre study. At present, we do not have long‐term outcome data. Nevertheless, patients were consecutive. Future prospective studies with larger cohorts across multiple centres are warranted further to validate these findings and to explore the long‐term outcomes of tenotomy performed by different healthcare professionals.

## 5 Conclusion

Podiatrist‐led percutaneous needle tenotomy is a safe and effective intervention for foot ulcers, achieving healing outcomes comparable to those reported for consultant orthopaedic surgeons, even in patients with more severe baseline disease.

Our findings support the role of podiatrists as key providers of minimally invasive surgical care within multidisciplinary diabetic foot teams. Expanding podiatrist‐led tenotomy services could enhance timely access to ulcer offloading procedures, potentially improving limb salvage rates. Larger prospective studies are needed to confirm these results and to define best practice guidelines across specialties.

## Author Contributions

All authors contributed equally to the work and the writing of this paper.

## Funding

The authors have nothing to report.

## Ethics Statement

This was a service evaluation, and the consent of the participating patients was recorded in the electronic patient hospital record. We thank the participants in the study.

## Conflicts of Interest

The authors declare no conflicts of interest.

## Data Availability

The datasets generated during and/or analyzed during the current study are available from the corresponding author on reasonable request.
